# Idiopathic portal hypertension in an "inactive" HBV carrier: a case report

**DOI:** 10.1186/1757-1626-1-229

**Published:** 2008-10-08

**Authors:** Themistoklis G Vassiliadis, Anthia Gatopoulou, Kaliopi Patsiaoura, Olga Giouleme, Konstantinos Soufleris, Alexandros Boubonaris, Panagiotis Katsinelos, Nikolaos Eugenidis

**Affiliations:** 1 2nd Propedeutic Clinic of Internal Medicine, Aristotle University of Thessaloniki, Greece; 2 Endoscopy Unit, Demokritus University of Thrace, Alexandroupolis, Greece; 3 Endoscopy and Motility Unit, Central Hospital, Thessaloniki, Greece

## Abstract

Idiopathic portal hypertension belongs to the group of non-cirrhotic portal hypertension, its etiology is still unknown but its prognosis is excellent. We report a case of 45 year old female with inactive hepatitis B virus (HBV) carrier status and persistently elevated alpha-fetoprotein (AFP), presented with features of portal hypertension and without evidence of cirrhosis or fibrosis on liver biopsy.

## Background

Inactive hepatitis B virus (HBV) carriers represent the largest group in chronic HBV infected patients [1].

Non-cirrhotic portal hypertension represents a heterogeneous group of diseases in which liver cirrhosis is not present and most often presented only with features of portal hypertension and not of parenchyma dysfunction [2]. Idiopathic portal hypertension (IPH) belongs to this category and is still one of the most important misdiagnoses of clinical practice. To inexperienced physicians presenting esophageal varices and upper gastrointestinal bleeding usually prompt an unfortunate diagnosis of cirrhosis. With the expectation of an excellent prognosis of IPH, a practicing gastroenterologist should be aware that "not all varices mean cirrhosis" [3]. In the present paper, we report a case of non-cirrhotic portal hypertension in patient with inactive HBV carrier status and persistently increased levels of AFP. To the best of our knowledge, there is no other such case reported in English literature.

## Case presentation

We report a case of a 45 year old Greek female (168 cm height, 62 kg weight), HBV inactive carrier, with thrombocytopenia, diagnosed 26 years ago when she was investigated due to bruises and petechchiae on both legs. She has no previous medical or family history. She does not smoke and reports neither drug nor alcohol abuse. She is the mother of two daughters.

Laboratory tests revealed only low platelets (PLT: 98.000/mm^3^). She was HbsAg, anti HBc and anti HBe positive, with normal myelogramm, suggesting secondary hypersplenism.

Repeating tests and close follow up over the years revealed the same HBV status, normal liver function tests (LFTs) and low levels of PCR HBV DNA with maximum level of 7.4 × 10^2 ^IU/ML and AFP 42–56 ng/ml persistently elevated since April 2004.

Upper gastrointestinal endoscopy revealed gastric varices type II without signs of bleeding and propranolol was prescribed to her.

Repeating imaging tests such as CT, MRI scanning, Doppler ultrasound and MR angiography confirmed the absence of portal or splenic vein thrombosis.

Histological evaluation of liver biopsy concluded that the overall histological findings were compatible with inactive HBV carrier state without evidence of any excess inflammation or features suggesting fibrosis or cirrhosis. Immunohistochemistry confirmed HbsAg presence and HbcAg absence. The reticulin stain was negative.

Further investigation did not prove any abnormality regarding prothrombotic conditions, autoantibody screening (including antiphosholipid), thyroid function. Levels of a1- antitrypsin, ferritin, and ceruloplasmin were normal. Serology excluded other viral coinfection with HDV or HCV. Schistosomiasis was not found. Since diagnosis, patient's condition has been stable and her course, so far, uneventful.

## Discussion

Portal hypertension in chronic hepatitis is thought to develop during progression of the chronic hepatitis to cirrhosis [4]. While the prognosis of the inactive HbsAg carrier state is usually benign [5].

Multiple episodes of reactivation or sustained reactivation can cause progressive hepatic damage and even decompensation [6].

Non-cirrhotic portal hypertension is the general term for a heterogeneous group of diseases that are due to intrahepatic or extrahepatic etiologies [7].

Idiopathic portal hypertension belongs to this group and is a most confusing and complex disorder [3]. It may be diagnosed in a patient with symptoms of portal hypertension (varices), patent portal and splenic veins and in the absence of signs of liver cirrhosis, or when no other reason can be found for liver disease [8].

There are several theories regarding its pathogenesis, unfortunately none have proved to be a single factor fully explaining the pathogenesis. These theories include the trace element-chemical theory, autoimmune theory, infection thrombosis and genetic theory [3].

In our case, the patient belongs to this group and close monitoring for the past 26 years has ruled out reactivation. She presented with thrombocytopenia and splenomegaly. Gastric varices and patent portal and splenic veins detected but with no liver damage. However, it is questioned whether the fact that she is an inactive HBV carrier could be ignored.

It is also hard to say whether her portal hypertension and inactive HBV carrier status is a coincidence. On the other hand, it is difficult to prove the case that an immunity connection between the two conditions (with unknown mechanism) could be the primary underlying disorder.

It is however widely accepted that autoimmune diseases (especially connective tissue diseases) increase the prevalence of idiopathic portal hypertension in certain patient groups [9].

In our patient neither an autoimmune condition nor thrombophillic factors (protein c, s deficiency factor V mutation) were found despite detailed investigation. Drugs, alcohol, medication and toxins were excluded by patient's history. Chronic exposure to arsenic or vinyl chemicals even if suspected could not be proven.

One paper reports two cases of women with chronic hepatitis C who developed severe thrombocytopenia due to portal hypertension without cirrhosis, indicating that portal hypertension might precede the onset of cirrhosis in type C hepatitis [4].

Moreover, it is worth mentioning that despite the elevated serum AFP (i.e. ≥ 10 ng/L) our patient has not so far developed either hepatocellular (HCC) or any other extrahepatic cancer.

Elevated serum AFP has however been associated with various chronic liver diseases and hepatic regeneration, apart from HCC [10]. The presence of HBsAg was related to elevated AFP, in one old study, but no abnormal AFP was found among inactive HBV carriers [11]. Mild elevation of AFP has also been found to correlate with viral hepatitis especially hepatitis C [12,13]. One case report described a 38 year old healthy man with persistence of AFP due to autosomal dominant inheritance pattern [14]. We were not able to confirm hereditary persistence of AFP in our case because we could not analyze AFP levels in all members in three generations of her family.

Our patient does not have chronic hepatitis and has not developed cirrhosis after 26 years of diagnosis and close follow up. To the best of our knowledge this is the first case of inactive HBV carrier and persistently elevated AFP presented with non cirrhotic portal hypertension in English literature. It would be useful for the physicians to be aware of such a possibility in similar cases despite its extremely rare incidence. Whether there is a causal relationship between the two conditions or non-cirrhotic portal hypertension might precede the onset of serious liver damage in type B hepatitis in our case remains to be seen.

## Consent

Written informed consent was obtained from the patient for publication of this case report and accompanying images. A copy of the written consent is available for review by the Editor- in Chief of this journal.

## Competing interests

The authors declare that they have no competing interests.

## Authors' contributions

VT analysed, interpreted the data and revised the manuscript. GA was a major contributor in writing the manuscript and analysed, interpreted the data. PK performed the histological examination and gave final approval. GO, SK and MA performed the endoscopies and revised the article. KP and EN gave final approval and revised the article.
